# *N*-(4-Meth­oxy­benzo­yl)benzene­sulfon­amide

**DOI:** 10.1107/S1600536814001330

**Published:** 2014-01-22

**Authors:** S. Sreenivasa, M. S. Nanjundaswamy, S. Madankumar, N. K. Lokanath, E. Suresha, P. A. Suchetan

**Affiliations:** a Department of Studies and Research in Chemistry, Tumkur University, Tumkur, Karnataka 572 103, India; b Department of Chemistry, AVK College for Women, Davangere-2, India; c Department of Studies in Physics, University of Mysore, Manasagangotri, Mysore, India; d University College of Science, Tumkur University, Tumkur, India; e Department of Studies and Research in Chemistry, U.C.S., Tumkur University, Tumkur, Karnataka 572 103, India

## Abstract

In the title compound, C_14_H_13_NO_4_S, the dihedral angle between the aromatic rings is 69.81 (1)°; the dihedral angle between the planes defined by the S—N—C=O segment of the central chain and the sulfonyl benzene ring is 74.91 (1)°. In the crystal, the mol­ecules are linked by weak N—H⋯O hydrogen bonds into *C*(4) chains running along [100]. The mol­ecules in adjacent chains are linked by weak C—H⋯O inter­actions, generating *R*_2_^2^ (16) dimeric pairs. Weak C—H⋯π inter­actions connect the double chains into (001) sheets.

## Related literature   

For similar structures, see: Gowda *et al.* (2009 ▶); Suchetan *et al.* (2009 ▶, 2010 ▶); Sreenivasa *et al.* (2013 ▶).
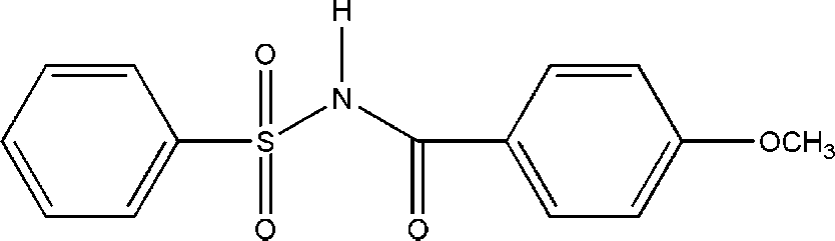


## Experimental   

### 

#### Crystal data  

C_14_H_13_NO_4_S
*M* *_r_* = 291.31
Triclinic, 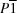

*a* = 5.3059 (5) Å
*b* = 10.6343 (10) Å
*c* = 11.9139 (11) Å
α = 89.792 (3)°
β = 87.392 (3)°
γ = 83.944 (3)°
*V* = 667.79 (11) Å^3^
*Z* = 2
Cu *K*α radiation
μ = 2.28 mm^−1^
*T* = 293 K
0.37 × 0.26 × 0.20 mm

#### Data collection  

Bruker APEXII diffractometer
Absorption correction: multi-scan (*SADABS*; Bruker, 2009 ▶) *T*_min_ = 0.515, *T*_max_ = 0.633
8561 measured reflections
2168 independent reflections
2076 reflections with *I* > 2σ(*I*)
*R*_int_ = 0.049

#### Refinement  

*R*[*F* ^2^ > 2σ(*F* ^2^)] = 0.068
*wR*(*F* ^2^) = 0.158
*S* = 1.13
2168 reflections
186 parameters
1 restraint
H atoms treated by a mixture of independent and constrained refinement
Δρ_max_ = 0.33 e Å^−3^
Δρ_min_ = −0.84 e Å^−3^

### 

Data collection: *APEX2* (Bruker, 2009 ▶); cell refinement: *APEX2* and *SAINT-Plus* (Bruker, 2009 ▶); data reduction: *SAINT-Plus* and *XPREP* (Bruker, 2009 ▶); program(s) used to solve structure: *SHELXS97* (Sheldrick, 2008 ▶); program(s) used to refine structure: *SHELXL97* (Sheldrick, 2008 ▶); molecular graphics: *Mercury* (Macrae *et al.*, 2008 ▶); software used to prepare material for publication: *SHELXL97*.

## Supplementary Material

Crystal structure: contains datablock(s) I. DOI: 10.1107/S1600536814001330/hb7187sup1.cif

Structure factors: contains datablock(s) I. DOI: 10.1107/S1600536814001330/hb7187Isup2.hkl

Supporting information file. DOI: 10.1107/S1600536814001330/hb7187Isup3.cml

CCDC reference:

Additional supporting information:  crystallographic information; 3D view; checkCIF report

## Figures and Tables

**Table 1:** Hydrogen-bond geometry (Å, °) *Cg* is the centroid of the meth­oxy­benzene ring.

*D*—H⋯*A*	*D*—H	H⋯*A*	*D*⋯*A*	*D*—H⋯*A*
N1—H*N*1⋯O1^i^	0.83 (3)	2.41 (3)	3.1662 (3)	153
C12—H12⋯O2^ii^	0.93	2.58	3.286 (3)	133
C4—H4⋯*Cg* ^iii^	0.93	2.90	3.7396	150

Symmetry codes: (i) 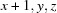; (ii) 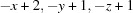; (iii) 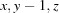.

**Fig. 1.**
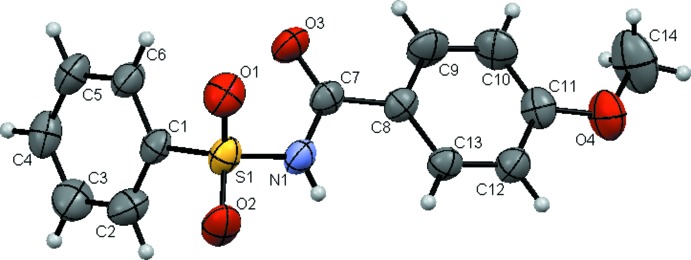
Molecular structure of the title compound, showing displacement ellipsoids drawn at the 50% probability level.

**Fig. 2.**
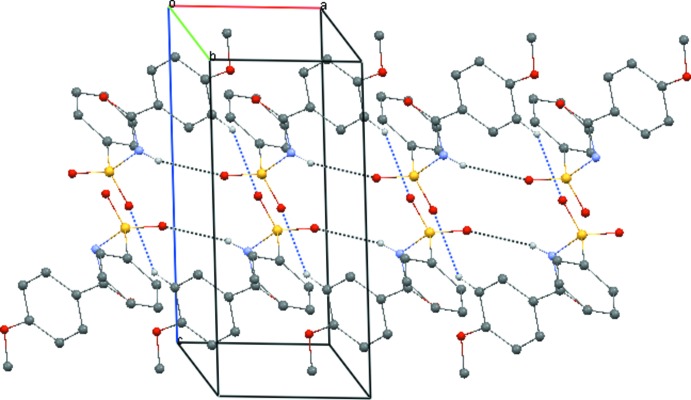
Columns generated from the combination of N1—HN1···O1 linked chains and C12—H12···O2 linked dimers in (I) when viewed along a axis. H-atoms not involved in H-bonds are omitted for clarity purpose.

**Fig. 3.**
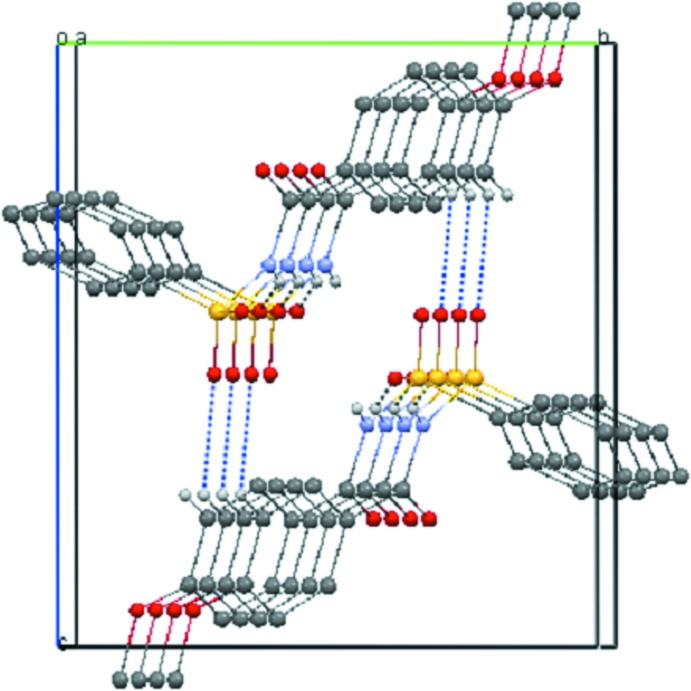
Columns generated from the combination of N1—HN1···O1 linked chains and C12—H12···O2 linked dimers in (I) when viewed down a axis. H-atoms not involved in H-bonds are omitted for clarity purpose.

**Fig. 4.**
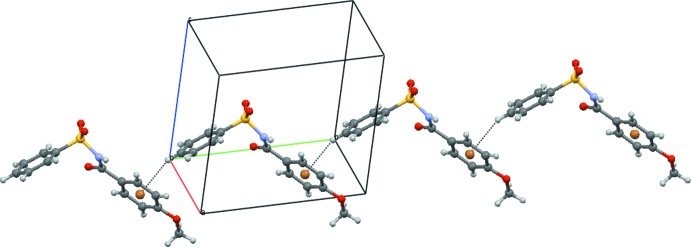
C4—H4···Cg interaction observed in the structure of (I). Cg is the centroid of the methoxyphenyl ring.

## supplementary crystallographic information

### 1. Introduction

As a part of our continued efforts to study the crystal structures of N-(aroyl)-aryl­sulfonamides (Suchetan *et al.*, 2009, 2010; Sreenivasa *et al.*, 2013; Gowda *et al.*, 2009), we report here the crystal structure of the title compound (I) (Fig 1).

### 2.1. Synthesis and crystallization

The title compound (I) was prepared by refluxing a mixture of 4-meth­oxy­benzoic acid, benzene­sulfonamide and phospho­rous oxychloride (POCl_3_) for 2 h on a water bath. The resultant mixture was cooled and poured into ice cold water. The solid obtained was filtered and washed thoroughly with water and then dissolved in sodium bicarbonate solution. The compound was later reprecipitated by acidifying the filtered solution with dilute HCl. The compound obtained was filtered and later dried (Melting point: 399 K).

Colorless prisms of (I) were obtained from a slow evaporation of its aqueous methano­lic solution at room temperature.

### 2.2. Refinement

The H atom of the NH group was located in a difference map and later restained to N—H = 0.86 (4) Å. The other H atoms were positioned with idealized geometry using a riding model with C—H = 0.93-0.96 Å. All H atoms were refined with isotropic displacement parameters (set to 1.2-1.5 times of the U eq of the parent atom).

### 3. Results and discussion

In (I), the dihedral angle between the aromatic rings is 69.81 (1)°. Compared to this, the dihedral angle is 80.3 (1)° in N-(benzoyl)-benzene­sulfonamide (II) (Gowda *et al.*, 2009), 68.6 (1)° in N-(4-chloro­benzoyl)-benzene­sulfonamide (III) (Suchetan *et al.*, 2009), 71.9 (1)° in N-(4-methyl­benzoyl)-benzene­sulfonamide (IV) (Suchetan *et al.*, 2010) and 78.62 (16)° in N-(4-meth­oxy­benzoyl)-4-methyl­benzene­sulfonamide (V) (Sreenivasa *et al.*, 2013). This shows that introducing a substituent into the *para* position of the benzoyl ring correlates with a decrease of the dihedral angle between the aromatic rings. Further, the molecule is twisted at the S atom, the dihedral angle between the planes defined by the S—N—C=O segment in the central chain and the sulfonyl benzene ring being 74.91 (1)°.

In the crystal, both strong classical N—H···O hydrogen bonds and weaker C—H···O and C—H···Cg inter­actions are observed. The molecules are linked to one another through strong N1—HN1···O1 hydrogen bonds into C(4) chains running along [100]. The molecules in the adjacent chains are linked through weak C12—H12···O2 inter­actions, generating R_2_^2^ (16) dimeric pairs. Combination of the N1—HN1···O1 linked chains and C12—H12···O2 linked dimers form columns along a *axis* (Figure 2 & 3). The molecules are further connected into C(10) chains running along [010] through C4—H4···Cg inter­actions (Figure 4) resulting in a two dimensional architecture.

### Crystal data

**Table:**

C_14_H_13_NO_4_S	*F*(000) = 304
*M**_r_* = 291.31	Prism
Triclinic, *P*1	*D*_x_ = 1.449 Mg m^−^^3^
Hall symbol: -P 1	Melting point: 399 K
*a* = 5.3059 (5) Å	Cu *K*α radiation, λ = 1.54178 Å
*b* = 10.6343 (10) Å	Cell parameters from 25 reflections
*c* = 11.9139 (11) Å	θ = 3.7–64.8°
α = 89.792 (3)°	µ = 2.28 mm^−^^1^
β = 87.392 (3)°	*T* = 293 K
γ = 83.944 (3)°	Prism, colourless
*V* = 667.79 (11) Å^3^	0.37 × 0.26 × 0.20 mm
*Z* = 2	

### Data collection

**Table:**

Bruker APEXII diffractometer	2076 reflections with *I* > 2σ(*I*)
Radiation source: fine-focus sealed tube	*R*_int_ = 0.049
Graphite monochromator	θ_max_ = 64.8°, θ_min_ = 3.7°
phi and φ scans	*h* = −6→6
Absorption correction: multi-scan (*SADABS*; Bruker, 2009)	*k* = −12→12
*T*_min_ = 0.515, *T*_max_ = 0.633	*l* = −13→13
8561 measured reflections	3 standard reflections every 120 min
2168 independent reflections	intensity decay: 1%

### Refinement

**Table:**

Refinement on *F*^2^	Primary atom site location: structure-invariant direct methods
Least-squares matrix: full	Secondary atom site location: difference Fourier map
*R*[*F*^2^ > 2σ(*F*^2^)] = 0.068	Hydrogen site location: inferred from neighbouring sites
*wR*(*F*^2^) = 0.158	H atoms treated by a mixture of independent and constrained refinement
*S* = 1.13	*w* = 1/[σ^2^(*F*_o_^2^) + (0.1013*P*)^2^ + 0.1754*P*] where *P* = (*F*_o_^2^ + 2*F*_c_^2^)/3
2168 reflections	(Δ/σ)_max_ < 0.001
186 parameters	Δρ_max_ = 0.33 e Å^−^^3^
1 restraint	Δρ_min_ = −0.84 e Å^−^^3^

### Special details

**Table:**

Geometry. All s.u.'s (except the s.u. in the dihedral angle between two l.s. planes) are estimated using the full covariance matrix. The cell s.u.'s are taken into account individually in the estimation of s.u.'s in distances, angles and torsion angles; correlations between s.u.'s in cell parameters are only used when they are defined by crystal symmetry. An approximate (isotropic) treatment of cell s.u.'s is used for estimating s.u.'s involving l.s. planes.
Refinement. Refinement of *F*^2^ against ALL reflections. The weighted *R*-factor *wR* and goodness of fit *S* are based on *F*^2^, conventional *R*-factors *R* are based on *F*, with *F* set to zero for negative *F*^2^. The threshold expression of *F*^2^ > 2σ(*F*^2^) is used only for calculating *R*-factors(gt) *etc*. and is not relevant to the choice of reflections for refinement. *R*-factors based on *F*^2^ are statistically about twice as large as those based on *F*, and *R*- factors based on ALL data will be even larger.

### Fractional atomic coordinates and isotropic or equivalent isotropic displacement parameters (Å^2^)

**Table:**

	*x*	*y*	*z*	*U*_iso_*/*U*_eq_	
O1	0.2427 (3)	0.36646 (17)	0.44504 (16)	0.0596 (5)	
N1	0.6766 (4)	0.40318 (19)	0.36803 (17)	0.0478 (5)	
O3	0.4534 (3)	0.39943 (16)	0.21164 (15)	0.0559 (5)	
O4	1.1816 (4)	0.81223 (19)	0.06023 (17)	0.0695 (6)	
C13	0.9563 (4)	0.5966 (2)	0.26610 (18)	0.0421 (5)	
H13	0.9898	0.5737	0.3398	0.051*	
C7	0.6197 (4)	0.4435 (2)	0.25993 (19)	0.0424 (5)	
O2	0.6259 (4)	0.30297 (18)	0.54898 (15)	0.0624 (5)	
C9	0.7269 (6)	0.5777 (3)	0.1010 (2)	0.0609 (7)	
H9	0.6030	0.5417	0.0631	0.073*	
C1	0.5310 (4)	0.1655 (2)	0.37956 (18)	0.0405 (5)	
C12	1.0878 (4)	0.6857 (2)	0.2130 (2)	0.0475 (6)	
H12	1.2101	0.7227	0.2512	0.057*	
C8	0.7732 (4)	0.5405 (2)	0.21008 (18)	0.0402 (5)	
C11	1.0410 (5)	0.7213 (2)	0.1032 (2)	0.0472 (6)	
C5	0.3772 (5)	0.0164 (2)	0.2584 (2)	0.0564 (6)	
H5	0.2579	−0.0048	0.2089	0.068*	
C3	0.7531 (5)	−0.0405 (3)	0.3552 (3)	0.0649 (8)	
H3	0.8890	−0.0996	0.3709	0.078*	
C4	0.5776 (5)	−0.0707 (2)	0.2829 (2)	0.0547 (6)	
H4	0.5930	−0.1504	0.2500	0.066*	
C2	0.7307 (5)	0.0778 (3)	0.4055 (2)	0.0574 (6)	
H2	0.8487	0.0979	0.4562	0.069*	
C14	1.1590 (10)	0.8451 (4)	−0.0546 (3)	0.0986 (13)	
H14A	1.2092	0.7720	−0.1005	0.148*	
H14B	1.2665	0.9100	−0.0734	0.148*	
H14C	0.9860	0.8757	−0.0676	0.148*	
C6	0.3516 (4)	0.1360 (2)	0.3071 (2)	0.0498 (6)	
H6	0.2157	0.1948	0.2910	0.060*	
C10	0.8596 (6)	0.6669 (3)	0.0467 (2)	0.0635 (7)	
H10	0.8267	0.6899	−0.0270	0.076*	
S1	0.50162 (10)	0.31426 (5)	0.44550 (4)	0.0452 (3)	
HN1	0.808 (6)	0.419 (4)	0.397 (3)	0.091 (12)*	

### Atomic displacement parameters (Å^2^)

**Table:**

	*U*^11^	*U*^22^	*U*^33^	*U*^12^	*U*^13^	*U*^23^
O1	0.0540 (10)	0.0592 (10)	0.0642 (11)	−0.0033 (8)	0.0092 (8)	−0.0138 (8)
N1	0.0543 (12)	0.0501 (11)	0.0432 (11)	−0.0222 (9)	−0.0093 (9)	0.0000 (8)
O3	0.0563 (10)	0.0581 (10)	0.0573 (10)	−0.0197 (8)	−0.0149 (8)	−0.0071 (8)
O4	0.0829 (14)	0.0677 (12)	0.0582 (12)	−0.0183 (10)	0.0158 (10)	0.0140 (9)
C13	0.0460 (12)	0.0463 (11)	0.0348 (11)	−0.0079 (9)	−0.0035 (9)	−0.0026 (9)
C7	0.0460 (12)	0.0400 (11)	0.0419 (12)	−0.0065 (9)	−0.0039 (9)	−0.0105 (9)
O2	0.0851 (13)	0.0689 (11)	0.0385 (9)	−0.0294 (10)	−0.0111 (9)	−0.0024 (8)
C9	0.0753 (18)	0.0729 (17)	0.0393 (13)	−0.0241 (13)	−0.0155 (12)	−0.0048 (11)
C1	0.0383 (11)	0.0445 (11)	0.0403 (11)	−0.0142 (8)	0.0036 (8)	−0.0022 (8)
C12	0.0477 (12)	0.0519 (13)	0.0445 (12)	−0.0126 (10)	−0.0019 (9)	−0.0006 (10)
C8	0.0427 (11)	0.0417 (11)	0.0361 (11)	−0.0047 (9)	0.0001 (9)	−0.0081 (8)
C11	0.0523 (13)	0.0460 (12)	0.0418 (12)	−0.0035 (10)	0.0118 (10)	−0.0010 (9)
C5	0.0568 (14)	0.0537 (14)	0.0624 (16)	−0.0215 (11)	−0.0058 (11)	−0.0110 (11)
C3	0.0542 (15)	0.0558 (15)	0.083 (2)	0.0029 (12)	−0.0004 (14)	−0.0073 (13)
C4	0.0572 (14)	0.0445 (12)	0.0628 (16)	−0.0153 (10)	0.0159 (12)	−0.0095 (10)
C2	0.0450 (13)	0.0652 (15)	0.0628 (15)	−0.0071 (11)	−0.0084 (11)	−0.0083 (12)
C14	0.147 (4)	0.091 (2)	0.0561 (19)	−0.018 (2)	0.032 (2)	0.0156 (17)
C6	0.0449 (12)	0.0469 (12)	0.0598 (14)	−0.0128 (9)	−0.0081 (10)	−0.0050 (10)
C10	0.084 (2)	0.0740 (17)	0.0338 (12)	−0.0134 (14)	−0.0043 (12)	0.0032 (11)
S1	0.0516 (4)	0.0468 (4)	0.0393 (4)	−0.0156 (3)	0.0011 (3)	−0.0074 (2)

### Geometric parameters (Å, º)

**Table:**

O1—S1	1.4264 (19)	C1—S1	1.758 (2)
N1—C7	1.392 (3)	C12—C11	1.387 (3)
N1—S1	1.646 (2)	C12—H12	0.9300
N1—HN1	0.82 (3)	C11—C10	1.376 (4)
O3—C7	1.211 (3)	C5—C4	1.376 (4)
O4—C11	1.367 (3)	C5—C6	1.390 (4)
O4—C14	1.418 (4)	C5—H5	0.9300
C13—C12	1.371 (3)	C3—C4	1.360 (4)
C13—C8	1.389 (3)	C3—C2	1.388 (4)
C13—H13	0.9300	C3—H3	0.9300
C7—C8	1.486 (3)	C4—H4	0.9300
O2—S1	1.4232 (19)	C2—H2	0.9300
C9—C10	1.381 (4)	C14—H14A	0.9600
C9—C8	1.382 (3)	C14—H14B	0.9600
C9—H9	0.9300	C14—H14C	0.9600
C1—C6	1.375 (3)	C6—H6	0.9300
C1—C2	1.381 (4)	C10—H10	0.9300
			
C7—N1—S1	123.91 (17)	C4—C3—C2	120.4 (2)
C7—N1—HN1	122 (3)	C4—C3—H3	119.8
S1—N1—HN1	114 (3)	C2—C3—H3	119.8
C11—O4—C14	118.2 (3)	C3—C4—C5	120.2 (2)
C12—C13—C8	120.1 (2)	C3—C4—H4	119.9
C12—C13—H13	119.9	C5—C4—H4	119.9
C8—C13—H13	119.9	C1—C2—C3	119.2 (2)
O3—C7—N1	120.0 (2)	C1—C2—H2	120.4
O3—C7—C8	123.6 (2)	C3—C2—H2	120.4
N1—C7—C8	116.42 (19)	O4—C14—H14A	109.5
C10—C9—C8	121.9 (2)	O4—C14—H14B	109.5
C10—C9—H9	119.1	H14A—C14—H14B	109.5
C8—C9—H9	119.1	O4—C14—H14C	109.5
C6—C1—C2	121.0 (2)	H14A—C14—H14C	109.5
C6—C1—S1	120.16 (18)	H14B—C14—H14C	109.5
C2—C1—S1	118.81 (18)	C1—C6—C5	118.7 (2)
C13—C12—C11	120.9 (2)	C1—C6—H6	120.6
C13—C12—H12	119.5	C5—C6—H6	120.6
C11—C12—H12	119.5	C11—C10—C9	119.1 (2)
C9—C8—C13	118.3 (2)	C11—C10—H10	120.4
C9—C8—C7	117.3 (2)	C9—C10—H10	120.4
C13—C8—C7	124.4 (2)	O2—S1—O1	119.44 (12)
O4—C11—C10	125.0 (2)	O2—S1—N1	103.74 (11)
O4—C11—C12	115.3 (2)	O1—S1—N1	109.21 (11)
C10—C11—C12	119.6 (2)	O2—S1—C1	108.88 (11)
C4—C5—C6	120.5 (2)	O1—S1—C1	108.65 (10)
C4—C5—H5	119.7	N1—S1—C1	106.11 (10)
C6—C5—H5	119.7		
			
S1—N1—C7—O3	−10.3 (3)	S1—C1—C2—C3	179.0 (2)
S1—N1—C7—C8	169.94 (15)	C4—C3—C2—C1	−1.4 (4)
C8—C13—C12—C11	0.0 (3)	C2—C1—C6—C5	−1.2 (4)
C10—C9—C8—C13	1.0 (4)	S1—C1—C6—C5	−178.49 (18)
C10—C9—C8—C7	−179.8 (2)	C4—C5—C6—C1	0.4 (4)
C12—C13—C8—C9	−0.6 (3)	O4—C11—C10—C9	−178.6 (2)
C12—C13—C8—C7	−179.8 (2)	C12—C11—C10—C9	0.1 (4)
O3—C7—C8—C9	−2.4 (3)	C8—C9—C10—C11	−0.7 (4)
N1—C7—C8—C9	177.3 (2)	C7—N1—S1—O2	−177.30 (19)
O3—C7—C8—C13	176.8 (2)	C7—N1—S1—O1	−48.9 (2)
N1—C7—C8—C13	−3.5 (3)	C7—N1—S1—C1	68.0 (2)
C14—O4—C11—C10	−6.6 (4)	C6—C1—S1—O2	153.0 (2)
C14—O4—C11—C12	174.7 (3)	C2—C1—S1—O2	−24.3 (2)
C13—C12—C11—O4	179.0 (2)	C6—C1—S1—O1	21.4 (2)
C13—C12—C11—C10	0.2 (4)	C2—C1—S1—O1	−155.9 (2)
C2—C3—C4—C5	0.6 (4)	C6—C1—S1—N1	−95.9 (2)
C6—C5—C4—C3	−0.1 (4)	C2—C1—S1—N1	86.8 (2)
C6—C1—C2—C3	1.7 (4)		

### Hydrogen-bond geometry (Å, º)

Cg is the centroid of the methoxybenzene ring.

**Table:**

*D*—H···*A*	*D*—H	H···*A*	*D*···*A*	*D*—H···*A*
N1—H*N*1···O1^i^	0.83 (3)	2.41 (3)	3.1662 (3)	153
C12—H12···O2^ii^	0.93	2.58	3.286 (3)	133
C4—H4···*Cg*^iii^	0.93	2.90	3.7396	150

Symmetry codes: (i) *x*+1, *y*, *z*; (ii) −*x*+2, −*y*+1, −*z*+1; (iii) *x*, *y*−1, *z*.
